# Model-Based Weaning Tests for VA-ECLS Therapy

**DOI:** 10.1155/2020/4503919

**Published:** 2020-04-06

**Authors:** Simon Habran, Thomas Desaive, Philippe Morimont, Bernard Lambermont, Pierre C. Dauby

**Affiliations:** ^1^University of Liège, GIGA-In Silico Medicine, Belgium; ^2^University of Liège, GIGA-Cardiovascular Sciences, Belgium

## Abstract

Venoarterial extracorporeal life support (VA-ECLS) is used in ICUs (intensive care units) for the most extreme presentations of acute and severe cardiogenic shock, and one of the main issues the clinicians have to deal with is the weaning from VA-ECLS. In this study, a patient-specific model of the cardiovascular system connected to a VA-ECLS is built to improve the understanding of this complex system. Pig experiments are performed to validate the model, and the results are quite promising since the mean difference between experimental data and simulation is smaller than 5% for all the hemodynamic quantities. It is also a major objective of this paper to provide a proof-of-concept analysis that model-based approaches could improve the weaning strategy for VA-ECLS therapy.

## 1. Introduction

Venoarterial extracorporeal life support (VA-ECLS) is usually applied in the clinical setting of the most extreme presentations of acute and severe cardiogenic shock considered refractory to traditional measures. This technique is generally used in ICUs (intensive care units) when it is the only remaining option to save a human life [1–3].

In current practice, the (arterial) injection cannula is introduced through the femoral artery in such a way that the tip of the cannula is placed in the distal aorta. A drainage (venous) cannula is inserted in the femoral vein (or, less frequently, in the jugular vein) and placed at the junction between the right atrium and inferior (or superior) vena cava. Even if VA-ECLS is nowadays commonly used in ICUs, the mortality rate associated with this device still remains high [2, 3]. Several improvements are thus required to decrease the complications associated with this extracorporeal device. One of the main issues the clinicians have to deal with is the weaning from VA-ECLS. Indeed, since this configuration drains a fraction of the systemic blood flow from the veins and returns it in the arteries, the VA-ECLS modifies the hemodynamics of the heart by decreasing the preload and increasing the afterload. The actual cardiac condition of a patient with VA-ECLS is thus not easy to assess, and in particular, it is difficult to predict how the heart will behave after the device is switched off. To try to answer this question before removing the VA-ECLS, the medical team performs several weaning tests by progressively decreasing the extracorporeal blood flow to approximately 0.5 l/min. With such a small extracorporeal blood flow, the interaction of the VA-ECLS with the heart becomes negligible and the quasireal hemodynamics of the heart can be evaluated by measuring the mean arterial pressure, the left ventricular ejection fraction, and the aortic velocity time integral [4]. However, low blood flows during weaning tests increase the risk of clotting.

Mathematical models of biological or physiological systems have proved their ability to provide a better understanding of the basic mechanisms at work in many complex situations. In particular, many mathematical models of the cardiovascular system have already been proposed and analyzed in literature [5–14]. However, only a few have studied the interactions between the cardiovascular system and an extracorporeal circulatory support device [5, 12]. In [5], Broomé and Donker have built a rather complex cardiovascular model connected to a VA-ECLS, with 32 compartments for the cardiovascular system and 1 compartment describing the ECLS. Given the usually rather low number of measurements available in the ICU, a rather large number of the many parameters of this model cannot be determined in a patient-specific way. On the other hand, Pironet et al. proposed a minimal model of the cardiovascular system, of which most (or all) parameters can be determined from data usually available in the ICU [15]. The first purpose of the present work is to build a similar model and complement it with an additional compartment describing the VA-ECLS. Of course, this extended model must remain identifiable with data available in the ICU. Then, the second and most important part of our work consists in showing in principle how such a model could help avoiding too low extracorporeal flows during weaning tests. This part of our work thus provides a proof-of-concept analysis of the possibility of model-based VA-ECLS weaning tests [16].

In the following sections, the mathematical model is briefly described and the experimental data that were obtained from 8 pigs are presented. Some parameters of the mathematical model can be estimated using the experimental data, and the method to compute these parameters is described. The hemodynamic quantities simulated by our model are then compared to the experimental data. In Section 4, a general discussion is presented. The values of the parameters and the comparison between the model and the experimental data are analyzed, and we explain that our work can be considered a first encouraging step towards the development of *in silico* weaning tests from a VA-ECLS. We also compare our simplified model with another model [5] presented in literature. Finally, a conclusion and possible future work are presented.

## 2. Methods

### 2.1. Mathematical Modeling

**Figure 1:**
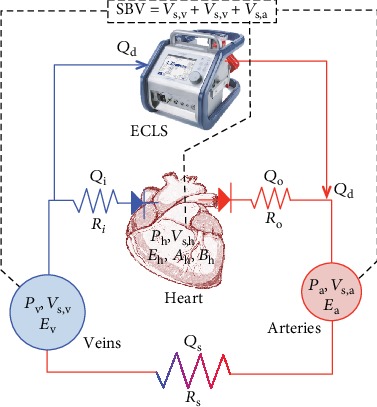
Model of the cardiovascular system connected to a VA-ECLS. All symbols are defined in the text.

Our model is a lumped parameter model of the cardiovascular system connected to a VA-ECLS. Beside the VA-ECLS, 3 other compartments are considered: one for the veins, one for the arteries, and one for the heart.  Figure 1 depicts a schematic representation of the 4-compartment model. The equations of the cardiovascular model will not be presented in detail here since they are quite similar to those introduced in Pironet et al. [15] (for the interested reader, these equations are briefly presented in Appendix A). Let us only mention that the artery compartment is characterized by the two variables *P*_a_ and *V*_s,a_ for the blood pressure and the stressed blood volume in the arteries and by one parameter *E*_a_ characterizing the elastance of the arteries. The vein compartment is characterized by the two variables *P*_v_ and *V*_s,v_ for the blood pressure and the stressed blood volume in the veins and by one parameter *E*_v_ which is the elastance of the veins. The heart compartment is characterized by the two variables *P*_h_ and *V*_s,h_ for the blood pressure and the stressed blood volume in the heart. The mathematical model also introduces 3 parameters to describe the heart compartment: the end-systolic elastance *E*_h_ is the slope of the end-systolic pressure-volume relationship, which characterizes the contractility of the heart (see Appendix A), and parameters *A*_h_ and *B*_h_ which describe the end-diastolic pressure-volume relationship (EDPVR, see Appendix A). Different blood flows must also be described. The blood flow *Q*_o_ is ejected by the heart and flows through a resistance *R*_o_ (the resistance of the output valve); the blood flow *Q*_s_, linking the arteries to the veins, flows through a resistance *R*_s_ (systemic resistance); and the blood flow *Q*_i_, which arrives in the heart, flows through a resistance *R*_i_ (resistance of the input valve). For the VA-ECLS device, only the extracorporeal blood flow *Q*_d_ which drains blood from the vena cava and returns it in the aorta is taken into account. Finally, parameter SBV is used to represent the total stressed blood volume in the cardiovascular system.

### 2.2. Experimental Data

Animal experiments, approved by the Ethics Committee of the Medical Faculty of the University of Liège, were carried out on 8 Pietrain pigs.

Pigs are administered ketamine (20 mg/kg) and diazepam (1 mg/kg) prior to the experiments. At the beginning of the experiments, the pigs are anesthetized by continuous infusion of sufentanil (0.5 *μ*g/kh/h) and sodium pentobarbital (3 mg/kg). The animals are intubated, and the ventilator (Engström Carestation, General Electric, Germany) is set to a tidal volume of 10 ml/kg, an inspired fraction of oxygen (*F*_I,O_2__) of 0.5, a respiratory frequency of 20 respirations per minute, and a PEEP (Positive End-Expiratory Pressure) of 5 cmH_2_O. Three catheters (Transonic, USA) are placed in the cardiovascular system: a conductance pressure-volume catheter is inserted in the left carotid artery and placed in the left ventricle, a pressure catheter with two sensors (the distance between the two sensors is equal to 40 cm) is inserted in the right femoral artery and placed in the aortic arch, and a pressure catheter with one sensor is inserted in the femoral vein and placed in the inferior vena cava. A Fogarty balloon is also placed in the inferior vena cava in order to allow the modification of the preload of the ventricle. Using the 3 catheters, the blood pressures in the left ventricle, in the aortic arch, in the vessel located 40 cm below the aortic arch (in the iliac artery), and in the inferior vena cava are continuously measured as well as the left ventricular volume. Furthermore, the two cannulae to be connected to the VA-ECLS are placed in the cardiovascular system as described in Introduction.

After introducing all the measurement instruments, the balloon and the cannulae, all the data are recorded and these initial values define the baseline situation. Then, a cardiac arrest is induced for 5 minutes by an electric current applied near the apex of the heart, leading to ventricular fibrillation. In addition, ventilation is stopped during this state. Then, the ventilator is restarted with the same settings as before except for the *F*_I,O_2__ which is increased to 1. In addition, a VA-ECLS (CARDIOHELP®, Getinge, Sweden) is connected to the cannulae and an extracorporeal blood flow is initiated. The settings of this extracorporeal device are such that the extracorporeal blood flow is as large as possible, with the constraint that the inlet pressure must remain larger than -60 mmHg. The gas flow through the ECLS is equal to 8 l/min, and the fraction of O_2_ in the gas entering the device is equal to 1. Furthermore, a defibrillator is used to restore a normal sinus rhythm. If the mean arterial blood pressure is smaller than 60 mmHg, noradrenaline is infused to restore a mean arterial blood pressure larger than this value.

When the cardiovascular system connected to the VA-ECLS is stabilized, weaning tests are performed with a procedure quite similar to the ones carried out on patients in the ICU. During the weaning tests, the extracorporeal blood flow is gradually decreased by approximately 15% every 5 min. At the end of each of these steps, the ventilator is turned off for 1 min to remove the effects of breathing on the circulatory system. During this phase, the experimental data are recorded. In the present study, the pressure in the aortic arch will not be used and it is rather the distal arterial pressure (the pressure in the iliac artery) that will be considered for the identification of the parameters. Indeed, the sensor in the aorta is close to the arterial cannula and blood reinjection could thus induce local perturbations in pressure measurements. Moreover, arterial pressure in the iliac artery is near identical to aortic pressure and can be considered a valuable surrogate to assess left ventricular afterload. Note also that in current clinical practice, arterial pressure is estimated using a peripheral arterial line, which thus provides information at some distance from the aorta, as in our experimental setting. The end of the weaning test is defined when the extracorporeal blood flow reaches approximately 0.3 l/min. As for the other steps, the extracorporeal blood flow is maintained for 5 min, the ventilator is turned off for 1 min, and the experimental data are recorded. Then, the extracorporeal blood flow is increased and fixed to its initial value (an extracorporeal blood flow as large as possible with the constraint of an inlet pressure larger than -60 mmHg). After at least 30 min of stabilization, a second weaning test is carried out for 6 animals.

### 2.3. Estimation of the Model Parameters

The heart rate (HR) and the extracorporeal blood *Q*_d_ are two parameters of the mathematical model which are directly measured in the lab. The estimation of the other parameters is more complex and is described now, first in the case of a given and fixed external blow flow *Q*_d_ and then in the case of a weaning test.

#### 2.3.1. Estimation of the Parameters for One Given Extracorporeal Blood Flow

The cardiovascular model connected to a VA-ECLS contains 9 parameters (*R*_i_, *R*_o_, *R*_s_, *B*_h_, *A*_h_, *E*_h_, *E*_a_, *E*_v_, and SBV), whose values must be determined, and we want to make these estimations as patient-specific as possible given the available data. In our experimental setting, these data are the continuous blood pressures in the arteries and veins, the stroke volume in the left ventricle, and the diastolic volume in the left ventricle. For the blood pressure in the arteries, two essential values are extracted: the mean blood pressure and the pulse blood pressure (or amplitude blood pressure). For the blood pressure in the veins, only the value of the mean blood pressure can be extracted. Indeed, this signal is not accurate enough to extract its amplitude. Consequently, the parameter *E*_v_ cannot be estimated since it depends strongly on this amplitude. In our study, this parameter will thus be considered constant and its value is taken from literature (Table 1). Three of the 8 remaining parameters must also be extracted from literature and considered constant since only 5 measurements are available (3 blood pressures and 2 blood volumes). These parameters are *R*_i_, *R*_o_, and *B*_h_ and their values are presented in Table 1. Parameters *R*_i_ and *R*_o_ are in fact nonidentifiable with the data normally available in the ICU (the blood pressure in the heart is often not measured in the ICU). In addition, except in the case of valve pathologies, the resistances of the valves should not vary much between different subjects or between different hemodynamic situations of a given subject. Then, the two parameters *B*_h_ and *A*_h_ that define the EDPVR equation cannot be both identified with the available data. In our study, we assume that parameter *A*_h_ is more likely to change since, in previous studies [6, 13], *B*_h_ is identical for both ventricles while *A*_h_ is smaller for the right ventricle than the left one. Therefore, *A*_h_ is taken as a patient-specific parameter, while *B*_h_ is taken from the literature (Table 1).

The remaining 5 parameters *R*_s_, *A*_h_, *E*_h_, *E*_a_, and SBV must thus be estimated from the data. In the following, the available data will be gathered in vector **y**^data^:

(1) $$\begin{array}{c} y^{\text{data}}=\left(\begin{array}{c} {\overset{¯}{P}}_{\text{a}}\\ PP_{\text{a}}\\ {\overset{¯}{P}}_{\text{v}}\\ \text{ma}{\text{x}}_{\text{T}}\left(V_{\text{h}}\right)\\ \text{SV} \end{array}\right), \end{array}$$

where

- ${\overset{¯}{P}}_{\text{a}}$ is the mean blood pressure in the arteries. This value is estimated with the following equation [17]: ${\overset{¯}{P}}_{\text{a}}=\left(1/3\right)\text{ma}{\text{x}}_{\text{T}}\left(P_{\text{a}}\right)+\left(2/3\right)\text{mi}{\text{n}}_{\text{T}}\left(P_{\text{a}}\right)$, where max_T_(*P*_a_) and min_T_(*P*_a_) are, respectively, the maximum and the minimum blood pressure in the arteries during one beat of duration *T*
- PP_a_ is the pulse blood pressure in the arteries. This value is estimated by subtracting the diastolic blood pressure in the arteries (min_T_(*P*_a_)) from the systolic blood pressure in the arteries (max_T_(*P*_a_)).
- ${\overset{¯}{P}}_{\text{v}}$ is the mean blood pressure in the veins. This value is estimated by calculating the mean venous pressure for one beat
- max_T_(*V*_h_) is the maximum blood volume in the heart during one cardiac period. This quantity is estimated using the pressure-volume catheter in the left ventricle
- SV is the stroke volume. SV is estimated using the pressure-volume catheter and by subtracting the minimum blood volume in the left ventricle (min_T_(*V*_h_)) from the maximum blood volume in the left ventricle (max_T_(*V*_h_))

Note that data are recorded on several cardiac beats and the 5 values in **y**^data^ are the means of the different quantities for the considered beats.

If **p** is the vector containing the 5 parameters, the parameter identification is performed by finding **p** = **p**^∗^ which minimizes the error function Ψ, defined as follows [15]:

(2) $$\begin{array}{c} \Psi \left(p\right)=\overset{5}{\underset{k=1}{\sum }}{\left(\frac{y_{k}^{\text{data}}-y_{k}^{\text{model}}\left(p\right)}{\sigma_{k}}\right)}^{2}, \end{array}$$

where

- **y**^model^(**p**) contains the same physiological quantities as **y**^data^ but these quantities are here determined by using the mathematical model for which the parameter values are defined by vector **p**
- *σ*_*k*_ are standard errors related to the different elements of vector **y**^data^ and defined as follows. For each weaning test and each given value of the extracorporeal blood flow, the experimental data are recorded on several cardiac beats. Then, the means and standard deviations corresponding to these beats and for the different elements of vector **y**^data^ are calculated. Finally, for any index *k* corresponding to an element of **y**^data^, the quantity *σ*_*k*_ is defined as the mean of the standard deviations introduced above, the mean being calculated over all the weaning tests carried out on our 8 pigs. These quantities allow to take into account the experimental errors in our lab, and their values are given in Table 2

#### 2.3.2. Estimation of the Parameters Corresponding to a Weaning Test

It is worth recalling that the main goal of our mathematical model is to simulate the behavior of the cardiovascular system during weaning tests, for which the extracorporeal blood flow is progressively decreased. However, if the extracorporeal blood flow decreases, it seems clear that the behavior of the cardiovascular system will change significantly. Indeed, for a reduced *Q*_d_, the preload and afterload of the heart are, respectively, decreased and increased, which modifies the behavior of the heart and consequently the behavior of the whole hemodynamics. The cardiovascular system has in fact several control and regulation systems to maintain or adapt important hemodynamic quantities like, for instance, the mean blood pressure. To take these controls into account in our approach, we will assume that the different parameters of the CVS depend on the imposed value of the external blood flow and to keep things as simple as possible, we will just consider the following linear relationships between the parameters and *Q*_d_:

(3) $$\begin{array}{c} \left\{\begin{array}{c} A_{\text{h}}=A_{\text{Sl},\text{h}}\cdot Q_{\text{d}}+A_{0,\text{h}},\\ E_{\text{h}}=E_{\text{Sl},\text{h}}\cdot Q_{\text{d}}+E_{0,\text{h}},\\ E_{\text{a}}=E_{\text{Sl},\text{a}}\cdot Q_{d}+E_{0,\text{a}},\\ R_{\text{s}}=R_{\text{Sl},\text{s}}\cdot Q_{d}+R_{0,\text{s}},\\ \text{SBV}=\text{SB}{\text{V}}_{\text{Sl}}\cdot Q_{\text{d}}+\text{SB}{\text{V}}_{0}, \end{array}\right. \end{array}$$

where *A*_Sl,h_, *A*_0,h_, *E*_Sl,h_, *E*_0,h_, *E*_Sl,a_, *E*_0,a_, *R*_Sl,s_, *R*_0,s_, SBV_Sl_, and SBV_0_ are 10 coefficients whose values must be determined from the available data. It is important to emphasize that these 10 coefficients do not have a straightforward physiological interpretation and they must in fact be considered a mathematical tool to describe in a phenomenological way the control mechanisms that the body uses to adapt the physiological parameters *A*_h_, *E*_h_, *E*_a_, *R*_s_, and SBV to changes of the extracorporeal flow *Q*_d_.

To estimate these 10 coefficients, the identification procedure introduced before must be modified as follows. Remind first that the extracorporeal blood flow is an input of the cardiovascular system connected to a VA-ECLS since the medical team can control this quantity. The identification of the 10 coefficients introduced above can thus be carried out using the same measurements as in the previous subsection but the measurements related to different extracorporeal blood flows *Q*_d_ will be taken into account and a new error function Ψ is introduced:

(4) $$\begin{array}{c} \Psi \left(c\right)=\overset{n}{\underset{j=1}{\sum }}\overset{5}{\underset{k=1}{\sum }}{\left(\frac{y_{k,j}^{\text{data}}-y_{k,j}^{\text{model}}\left(c\right)}{\sigma_{k}}\right)}^{2}, \end{array}$$

where *n* is the number of the different extracorporeal blood flows and **c** is the vector of the 10 sought coefficients. With this method, the number of measurements is multiplied by the number of extracorporeal blood flows used for the identification. Since 10 values must be identified, *n* must be larger than or equal to 2. In this study, the parameter identification is performed using *n* ≥ 3.

### 2.4. Statistics

In the minimization process allowing the identification of the 10 coefficients, the minimum value Ψ(**c**^∗^) of Ψ(**c**) is not exactly equal to 0 and there is thus an error between the experimental data and the corresponding values estimated by the mathematical model. It is interesting to analyze separately the errors corresponding to the different components of the vector defined in equation (1), and it is worth introducing the following quantities:

(5) $$\begin{array}{c} \overset{¯}{\left|\Delta \text{P}{\text{P}}_{\text{a}}/\text{P}{\text{P}}_{\text{a}}\right|}=\frac{1}{n}\overset{n}{\underset{j=1}{\sum }}\frac{\left|\text{P}{\text{P}}_{\text{a},j}^{\text{ sim}}-\text{P}{\text{P}}_{\text{a},j}^{\;\exp}\right|}{\text{P}{\text{P}}_{\text{a},j}^{\;\exp}},\\ \overset{¯}{\left|\Delta {\overset{¯}{P}}_{\text{a}}/{\overset{¯}{P}}_{\text{a}}\right|}=\frac{1}{n}\overset{n}{\underset{j=1}{\sum }}\frac{\left|{\overset{¯}{P}}_{\text{a},j}^{\text{sim}}-{\overset{¯}{P}}_{\text{a},j}^{\exp}\right|}{{\overset{¯}{P}}_{\text{a},j}^{\exp}},\\ \overset{¯}{\left|\Delta {\overset{¯}{P}}_{\text{v}}/{\overset{¯}{P}}_{\text{v}}\right|}=\frac{1}{n}\overset{n}{\underset{j=1}{\sum }}\frac{\left|{\overset{¯}{P}}_{\text{v},j}^{\text{sim}}-{\overset{¯}{P}}_{\text{v},j}^{\exp}\right|}{{\overset{¯}{P}}_{\text{v},j}^{\exp}},\\ \overset{¯}{\left|\frac{\Delta \text{ma}{\text{x}}_{\text{T}}\left(V_{\text{h}}\right)}{\text{ma}{\text{x}}_{\text{T}}\left(V_{\text{h}}\right)}\right|}=\frac{1}{n}\overset{n}{\underset{j=1}{\sum }}\frac{\left|\text{ma}{\text{x}}_{\text{T}}{\left(V_{\text{h},j}\right)}^{\text{sim}}-\text{ma}{\text{x}}_{\text{T}}{\left(V_{\text{h},j}\right)}^{\exp}\right|}{\text{ma}{\text{x}}_{\text{T}}{\left(V_{\text{h},j}\right)}^{\exp}},\\ \overset{¯}{\left|\Delta \text{SV}/\text{SV}\right|}=\frac{1}{n}\overset{n}{\underset{j=1}{\sum }}\frac{\left|\text{S}{\text{V}}_{j}^{\text{sim}}-SV_{j}^{\exp}\right|}{\text{S}{\text{V}}_{j}^{\exp}}, \end{array}$$

where $\overset{¯}{\left|\Delta \text{P}{\text{P}}_{\text{a}}/\text{P}{\text{P}}_{\text{a}}\right|}$ is the mean relative error between simulated and experimental PP_a_, while index *j* corresponds to the different values of *Q*_d_. The other symbols introduced in equations (6–9) have similar meanings. In order to estimate if the model tends to overestimate or underestimate the experimental data, the mean relative errors are also computed without the absolute values (and the corresponding quantities are denoted as $\overset{¯}{\Delta \text{P}{\text{P}}_{\text{a}}/\text{P}{\text{P}}_{\text{a}}}$, $\overset{¯}{\Delta {\overset{¯}{P}}_{\text{a}}/{\overset{¯}{P}}_{\text{a}}}$, $\overset{¯}{\Delta {\overset{¯}{P}}_{\text{v}}/{\overset{¯}{P}}_{\text{v}}}$, $\overset{¯}{\Delta \text{ma}{\text{x}}_{\text{T}}\left(V_{\text{h}}\right)/\text{ma}{\text{x}}_{\text{T}}\left(V_{\text{h}}\right)}$, and $\overset{¯}{\Delta \text{SV}/\text{SV}}$). These 5 errors are calculated for each weaning test, and the mean and standard deviation over all tests are also estimated.

## 3. Results

Here, we present the results of the identification of the parameters of the model from experimental data. Then, the predictions of the model are compared to observations and the errors between the simulated values and the corresponding experimental data are also estimated.

### 3.1. Values of the Parameters during Baseline Situation

During the baseline situation (this phase corresponds to the data recorded before the cardiac arrest, see Subsection 2.2), the parameter HR is directly measured and the 5 physical parameters of the mathematical model, *A*_h_, *E*_h_, *E*_a_, *E*_v_, and SBV, are estimated using the methods described in Subsection 2.3.1 for *Q*_d_ = 0 l/min. With this method and our experimental data, the errors between the simulated values and the measurements are very small (Ψ(**p**^∗^) ≤ 10^−2^ for all pigs) and the values of the 5 parameters and HR for each pig are given in Table 3. It is worth emphasizing that all the parameters have the same order of magnitude for the different pigs.

### 3.2. Evolution of the Parameters during the Weaning Tests

**Figure 2:**
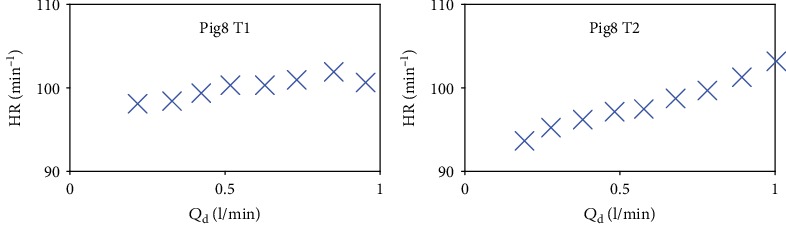
Heart rate in terms of *Q*_d_ for the weaning tests Pig8 T1 and Pig8 T2.

For the parameter identification during the weaning tests, HR and *Q*_d_ are directly measured and the values of HR in terms of *Q*_d_ for Pig8 are illustrated in Figure 2. For this pig, 2 weaning tests, labeled T1 and T2, have been performed.

The values of the 10 coefficients introduced in equation (3) and describing the evolution of the parameters with respect to *Q*_d_ are determined by the method presented in Subsection 2.3.2. Their values are provided in Table 4 for each weaning test. Note that the values of the independent terms in equation (3) are of the same order for the different weaning tests. However, the values of the slopes in equation (3) are quite different for the different pigs and even for different weaning tests corresponding to a given pig. Note also that Table 4 provides the possibly infused noradrenaline. In addition, Table 4 provides the number of *Q*_d_ that were used for each weaning test.

**Figure 3:**
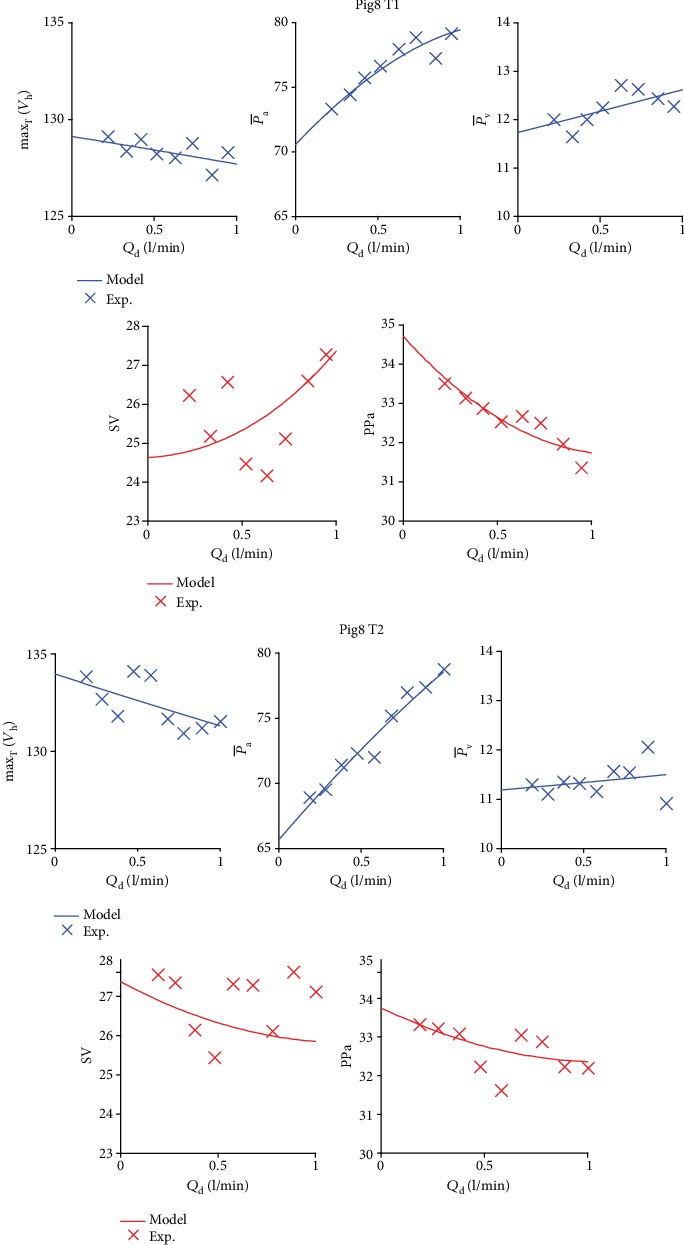
Comparison between experimental and simulated max_T_(*V*_h_), ${\overset{¯}{P}}_{\text{a}}$, ${\overset{¯}{P}}_{\text{v}}$, SV, and PP_a_ in terms of *Q*_d_ for the weaning test Pig8 T1 and Pig8 T2.

To assess the quality of the model, the hemodynamic variables obtained in simulations are compared with experimental data. As an example, Figure 3 shows a good agreement between the evolutions of simulated (solid lines) and experimental (crosses) physiological quantities of equation (1) for Pig8 and during the two weaning tests T1 and T2.

Table 5 describes the errors between the simulations and the experimental data. The relative mean errors are in fact quite small: for all the weaning tests, the quantities $\overset{¯}{\left|\Delta \text{P}{\text{P}}_{\text{a}}/\text{P}{\text{P}}_{\text{a}}\right|}$, $\overset{¯}{\left|\Delta {\overset{¯}{P}}_{\text{a}}/{\overset{¯}{P}}_{\text{a}}\right|}$, $\overset{¯}{\left|\Delta {\overset{¯}{P}}_{\text{v}}/{\overset{¯}{P}}_{\text{v}}\right|}$, and $\overset{¯}{\left|\Delta \text{ma}{\text{x}}_{\text{T}}\left(V_{\text{h}}\right)/\text{ma}{\text{x}}_{\text{T}}\left(V_{\text{h}}\right)\right|}$ are all smaller than 3% while $\overset{¯}{\left|\Delta \text{SV}/\text{SV}\right|}$ is smaller than 5%. Table 5 also shows that the simulated venous pressure overestimates the measured venous pressure ($\overset{¯}{\Delta {\overset{¯}{P}}_{\text{v}}/{\overset{¯}{P}}_{\text{v}}}=1.1\%$) while there is no global overestimation or global underestimation for the other quantities. For the interested reader, more details on the errors (errors for each weaning test) are provided in Table 6.

## 4. Discussion

### 4.1. Validation of Our Mathematical Models

Two models have been used in this study, one describing the cardiovascular system with no extracorporeal device (*Q*_d_ = 0) and one describing the cardiovascular system during the weaning of ECLS (several *Q*_d_).

The cardiovascular model alone has shown an excellent correspondence with experimental data since the estimated value of Ψ(**p**^∗^) is very small for all pigs. For the mathematical model simulating weaning tests, Table 5 has shown very small errors for PP_a_, ${\overset{¯}{P}}_{\text{a}}$, ${\overset{¯}{P}}_{\text{v}}$, and max_T_(*V*_h_) (relatives errors smaller or equal to 3%). The errors for SV are slightly larger (smaller than 5%) but still acceptable regarding the poor accuracy of SV measurements. Indeed, the mean value of SV is approximatively equal to 30 ml for all weaning tests and the corresponding measurement standard error is equal to 3 ml (see Table 2).

These results thus provide a convincing validation of our modeling approach.

### 4.2. Variations of the Parameters with *Q*_d_

Table 4 has provided the slopes of the 5 model parameters *E*_h_, SBV, *A*_h_, *E*_a_, and *R*_s_ with respect to *Q*_d_. These quantities are of paramount importance since they describe the variations of the parameters with the extracorporeal blood flow. These variations result from control mechanisms on the cardiovascular system when *Q*_d_ is changed and can thus be patient-specific.

In order to analyze these variations more deeply, it is interesting to introduce the following “normalized” changes of the 5 parameters when *Q*_d_ is increased from 0 to 1 l/min:

(6) $$\begin{array}{c} \Delta_{1}E_{\text{h}}/E_{\text{h}}=\frac{E_{\text{h}}\left({\text{Q}}_{\text{d}}=1\text{ l}/\min\right)-E_{\text{h}}\left({\text{Q}}_{\text{d}}=0\text{ l}/\min\right)}{E_{\text{h}}\left({\text{Q}}_{d}=0\text{ l}/\min\right)},\\ \Delta_{1}\text{SBV}/\text{SBV}=\frac{\text{SBV}\left(Q_{\text{d}}=1\text{ l}/\min\right)-\text{SBV}\left(Q_{\text{d}}=0\text{ l}/\min\right)}{\text{SBV}\left(Q_{\text{d}}=0\text{ l}/\min\right)},\\ \Delta_{1}A_{\text{h}}/A_{\text{h}}=\frac{A_{\text{h}}\left(Q_{\text{d}}=1\text{ l}/\min\right)-A_{\text{h}}\left(Q_{\text{d}}=0\text{ l}/\min\right)}{A_{h}\left(Q_{\text{d}}=0\text{ l}/\min\right)},\\ \Delta_{1}E_{\text{a}}/E_{\text{a}}=\frac{E_{\text{a}}\left(Q_{\text{d}}=1\text{ l}/\min\right)-E_{\text{a}}\left(Q_{\text{d}}=0\text{ l}/\min\right)}{E_{\text{a}}\left({\text{Q}}_{\text{d}}=0\text{ l}/\min\right)},\\ \Delta_{1}R_{\text{s}}/R_{\text{s}}=\frac{R_{\text{s}}\left(Q_{\text{d}}=1\text{ l}/\min\right)-R_{\text{s}}\left(Q_{\text{d}}=0\text{ l}/\min\right)}{R_{\text{s}}\left(Q_{\text{d}}=0\text{ l}/\min\right)}. \end{array}$$

**Figure 4:**
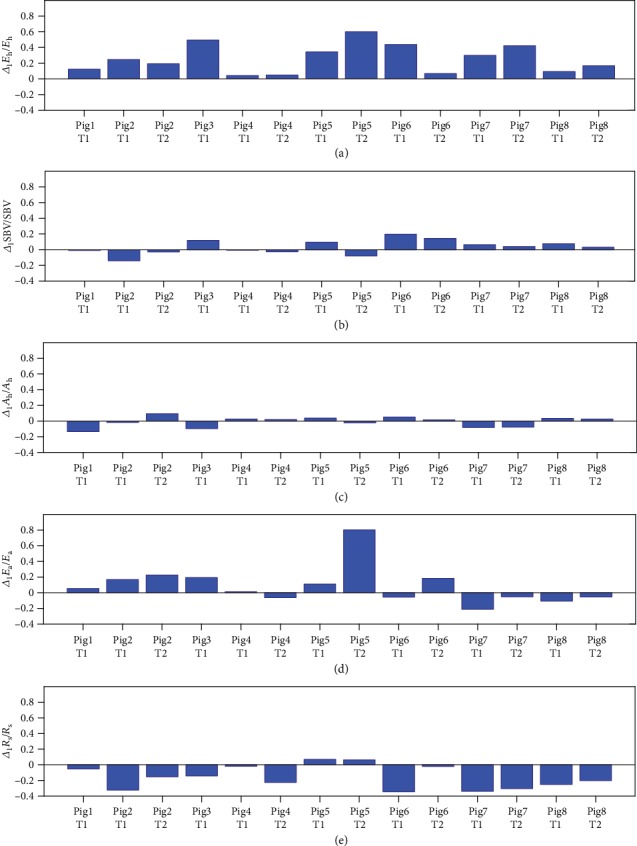
Values of Δ_1_*E*_h_/*E*_h_, Δ_1_SBV/SBV, Δ_1_*A*_h_/*A*_h_, Δ_1_*E*_a_/*E*_a_, and Δ_1_*R*_s_/*R*_s_ for each weaning test.

The values of these quantities are presented in Figure 4 for all the weaning tests. The first panel of the figure represents Δ_1_*E*_h_/*E*_h_ and demonstrates that Δ_1_*E*_h_/*E*_h_ is always positive. This implies that *E*_h_ always decreases when *Q*_d_ decreases. Such a change of the contractility with *Q*_d_ is not surprising since the preload and the afterload change with *Q*_d_. Indeed, the load independence of the ventricular contractility is only true for an instantaneous variation of the PV loop [18, 19]. In the situation analyzed in our work, the preload and afterload are changed for a relatively long period (approximately 5 min between 2 different *Q*_d_) and the heart has time enough to adjust its activity. Figures 4(b) and 4(c) show that the variations Δ_1_SBV/SBV and Δ_1_*A*_h_/*A*_h_ are small in comparison with Δ_1_*E*_h_/*E*_h_ and that the sign of the variations changes across the different animals and weaning tests, which reveal patient-specific reactions. Figure 4(d) demonstrates that parameter *E*_a_ varies significantly and that the sign of Δ_1_*E*_a_/*E*_a_ changes across the different animals and weaning tests. The evolution of this quantity is thus also patient-specific. Finally, Figure 4(e) describes Δ_1_*R*_s_/*R*_s_. This graph demonstrates that, except for Pig5, Δ_1_*R*_s_/*R*_s_ is always negative. Parameter *R*_s_ thus increases when *Q*_d_ decreases. This result could possibly be related to the baroreflex. Indeed, when *Q*_d_ decreases, the aortic pressure decreases and, in order to restore a higher blood pressure in the arteries, the nervous system constricts small vessels, which increases *R*_s_.

### 4.3. Values of the Parameters for *Q*_d_ = 0

It is very interesting to compare the values of the 5 model parameters *E*_h_, SBV, *A*_h_, *E*_a_, and *R*_s_ in the baseline situation to the values obtained from equation (3) with *Q*_d_ = 0. First, the data set in Table 3 describes the animal initial healthy cardiovascular status. Then, the first 5 lines of Table 4 can be considered the model-based estimation of the animal cardiovascular status if the VA-ECLS is switched off after the weaning test. This status is of course pathological since a 5 min cardiac arrest has been induced before starting the VA-ECLS therapy. Moreover, the status of the animal is also influenced by the possible side effects of the VA-ECLS therapy and by the injection of noradrenaline to some animals (see penultimate line of Table 4), which is known to increase the heart contractility and to constrict blood vessels.

The mean SBV, *A*_h_, and *E*_a_ for all the pigs during the baseline situation are relatively similar to, respectively, the mean SBV_0_, *A*_0,h_, and *E*_0,a_ during ECLS therapy (compare the last columns of Tables 3 and 4). On the other hand, the mean *E*_h_ during the baseline situation is larger than the mean *E*_0,h_. The two tables also show that the decrease in *E*_h,0_ is even more significant when there is no noradrenaline injection during the weaning test (see Pig1 T1, Pig5 T1, Pig7 T1, Pig7 T2, Pig8 T1, and Pig 8 T2). The decrease of the end-systolic elastance of the heart after the baseline situation corresponds to a reduction of the heart contractility and can be considered a consequence of the imposed cardiac arrest which has damaged the heart. In addition, this interpretation is strongly reinforced by the analysis of the noradrenaline role, which clearly limits the decrease in contractility for Pig1 T1, Pig5 T1, Pig7 T1, Pig7 T2, Pig8 T1, and Pig 8 T2.

It is also interesting to mention that several studies [20, 21] have demonstrated that extracorporeal circulations, such as VA-ECLS, can induce septic shocks and this pathology is known to decrease systemic resistance *R*_s_. Tables 3 and 4 confirm this effect since the mean *R*_s_ during the baseline situation is a little bit larger than the mean *R*_s,0_.

### 4.4. Application in the ICU and Model-Based Weaning Tests

The hemodynamic status and the cardiac condition of a patient with VA-ECLS must be assessed carefully before removing the device. As explained in Introduction, the weaning tests performed in ICUs are aimed at determining how the patient would behave without the VA-ECLS, *i.e.*, at *Q*_*d*_ = 0, but the process of decreasing the extracorporeal flow at low values is delicate and it would be desirable to predict what would happen at zero *Q*_d_ before actually decreasing the external flow to too low values.

A model-based weaning test using our modeling approach would consist in identifying the model parameters and coefficients at not too small values of *Q*_d_, which is a safe procedure for the patient, and using the model equations to predict the hemodynamic status of the patient if the VA-ECLS were removed. As explained in the previous section, our model can indeed provide, for instance, estimates at zero extracorporeal flow for the end-systolic elastance and the systemic resistance, which are two very important parameters characterizing a patient's hemodynamic status. More precisely, let us recall that in the ICU, commonly accepted conditions for weaning from VA-ECLS are the following. If a patient with a VA-ECLS, who has recovered from its major metabolic disturbance, has a pulsatile arterial waveform for at least 24 hours and has a mean arterial pressure higher than 60 mmHg without high levels of catecholamine, clinicians can proceed to a weaning test [4]. The test consists in gradually decreasing the extracorporeal blood flow and checking if the following 3 conditions on the systemic hemodynamics are satisfied when this flow has reached approximately 0.5−1 l/min [4]:

1. VTI > 12 cm (VTI is the velocity time integral and it is proportional to SV (SV = *π*(*ϕ*_LVOT_/2)^2^VTI, where VTI is the velocity time integral of flow through the left ventricular outflow tract (LVOT) and *ϕ*_LVOT_ is the diameter of the LVOT. These values can be estimated using Doppler echocardiography.))
2. Left ventricular ejection fraction (described in our model by *S*V/max_T_(*V*_h_)) > 20 − 25%
3. Mean arterial blood pressure (${\overset{¯}{P}}_{\text{a}}$) remains >60 mmHg

Since the stroke volume, ejection fraction, and mean arterial pressure can be calculated by our model, the 3 conditions above could be analyzed *in silico*, and the decision of removing the VA-ECLS could be made without decreasing *Q*_d_ to potentially dangerous values. All this argument thus indicates that our work can actually be considered an important first step towards model-based and safer weaning from a VA-ECLS.

### 4.5. Comparison with [5]

Broomé and Donker have built a 32-compartment model connected to a VA-ECLS [5], and the authors have compared their simulations with the experimental data provided in a work by Ostadal *et al.* [22]. In the present section, we use our 3-compartment model to describe the same experimental data and we compare our approach with that of Broomé *et al.* The data in [22], which can be used in our model, consists of the following vector, whose time evolution is provided for 5 extracorporeal blood flows (1, 2, 3, 4, and 5 l/min):

(7) $$\begin{array}{c} y_{\text{Ostadal}}^{\text{data}}=\left(\begin{array}{c} \text{SBP}\\ \text{EDP}\\ \text{EDV}\\ \text{SV} \end{array}\right), \end{array}$$

where SBP, EDP, EDV, and SV are, respectively, the systolic and end-diastolic blood pressures in the left ventricle, the end-diastolic volume in the left ventricle, and the stroke volume. Only 4 experimental physiological quantities are available in the data, and we can thus identify only 4 out the 5 parameters of our model. Moreover, since no information is provided on the diastolic blood pressure in the arteries, *E*_a_ is not determined from the data and this parameter is given a fixed value obtained from the literature (*E*_a_ = 0.76 mmHg/ml [6]). The remaining 4 parameters *R*_s_, *A*_h_, *E*_h_, and SBV can then be estimated from the experimental data. First, we estimate these parameters using the method presented in Subsection 2.3.1, which does not allow a change of the parameters when *Q*_d_ is varied. Then, we also use the approach which is introduced in Subsection 2.3.2 and which is based on a linear dependence of the physiological parameters with respect to *Q*_d_ (see equation (3) for *R*_s_, *A*_h_, *E*_h_, and SBV).

**Figure 5:**
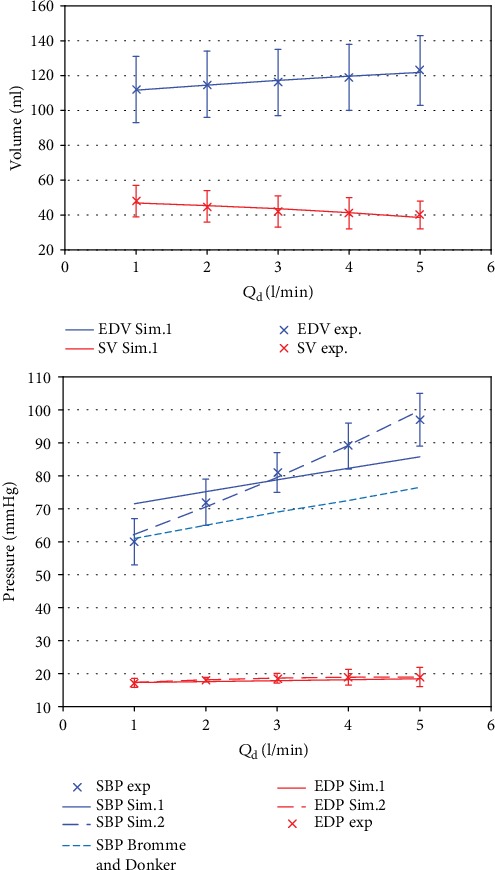
Comparison between experimental (crosses) and simulated (solid, dashed, and dotted lines) hemodynamic quantities in terms of *Q*_d_. “Sim. 1” and “Sim. 2” refer to our simulations with, respectively, *Q*_d_-independent and *Q*_d_-dependent physiological parameters, while “Brommé and Donker” indicates the results of the simulations in [5]. “Exp” refers to the experimental data of [22].

In the context of the first approach of Subsection 2.3.1, with *Q*_d_-independent physiological parameters, we have identified *R*_s_ = 0.456 mmHg · s/ml, *A*_h_ = 0.037 ml^−1^, *E*_h_ = 1.075 mmHg/ml, and SBV = 843 ml using the experimental data corresponding to an extracorporeal blood flow of 3 l/min.  Figure 5 presents the associated results and compares our simulations (solid lines, “Sim. 1”) with the measured quantities (crosses). This figure shows a good agreement between experimental and simulated EDP, EDV, and SV plotted as functions of *Q*_d_. On the other hand, the increase of SBP with *Q*_d_ is underestimated by our model (note that the SBP value for *Q*_d_ = 3 l/min is nicely described because the physiological parameters were identified for that value of the extracorporeal blood flow). In Figure 5, the dotted line reproduces the results of Broomé and Donker (extracted from their Figure 1). It is worth emphasizing that the parameters of the 32-compartment model of Broomé and Donker are also *Q*_d_-independent and we see from Figure 5 that the slope of their SBP–*Q*_d_ curve is also underestimated and quite similar to ours. This result was emphasized by Broomé and Donker, who suggest that the experimental steeper increase of SBP is due to an increase of the systolic vascular resistance (*R*_s_ in our model) and this increase of *R*_s_, which is not accounted for in the modeling approach, may be explained by myogenic vascular autoregulation or altered anesthetic depth.

To account for the control mechanisms in the body, we can use the approach presented in Subsection 2.3.2 and introduce in our model the *Q*_d_-dependence of the physiological parameters. The assumed linear variations of the parameters are described by the 8 coefficients *A*_Sl,h_, *A*_0,h_, *E*_Sl,h_, *E*_0,h_, *R*_Sl,s_, *R*_0,s_, SBV_Sl_, and SBV_0_, which can be estimated using the data (7) corresponding to the 5 *Q*_d_.  Table 7 shows the values of these coefficients and also provides the “normalized” changes (6) of the 4 parameters. As suggested by Broomé and Donker, the value of *R*_s_ actually increases with *Q*_d_. It is interesting to emphasize that Table 7 shows that the contractility *E*_h_ also increases significantly with *Q*_d_, which is an effect that we have also observed with our own experiments (see Subsection 2.3.2). In Figure 5, our results with *Q*_d_-dependent physiological parameters are also represented (dashed lines, “Sim. 2”) and compared with the data of Ostadal et al. and with the two models considered above. From this figure, we observe that accounting for the control mechanims in the body provides a nice description of the SBP–*Q*_*d*_ curve, with a correct slope.

From the comparison provided by Figure 5, one can conclude that the 32 compartments of the Broomé and Donker. model are not strictly required to simulate a weaning test. Our much simpler model, with only 3 compartments, can also perform very well and even describe better some physiological quantities if the control mechanisms of the body are introduced in the picture. In particular, this comparison also shows that modeling the pulmonary circulation does not seem needed to simulate weaning tests for VA-ECLS therapy.

### 4.6. Limitations of Our Approach

Several assumptions were made to build the mathematical model of the cardiovascular system connected to a VA-ECLS. An important hypothesis is the simplicity of the model. However, since few measurements are usually available in the ICU, the complexity of patient-specific mathematical model cannot be increased much. Let us also emphasize that right-left ventricular interaction is not considered in our approach, even if this can be sometimes a key point in weaning tests, especially in the case of right heart failure. This situation can thus not be analyzed with our model and would require a more complex approach.

Note also that left ventricle volume measurements are used for the parameter identification. These measurements are known to be not very accurate, which can influence the precision of some estimated parameters. A potential and partial solution, which could improve the estimation of stroke volume, could be to take an additional continuous measurement of cardiac output by using an ultrasound flow probe surrounding the aorta [23].

It is then important to emphasize that the weaning tests performed on the 8 pigs were undertaken in conditions different from those corresponding to ICUs. Indeed, for the pigs, the weaning tests began approximately one hour after the cardiac arrest, while for ICU patients, weaning tests can be considered after at least 24 hours of stable hemodynamic status. The animals considered in our work are thus less stable and still have heart failure symptoms during the weaning tests. In addition, the vessels of the studied pigs are smaller than those of adult humans and the range of extracorporeal blood flows is thus rather limited in the pig experiments. In order to overcome these limitations, additional experiments should be carried out on larger animals and last longer.

Finally, measurements of stroke volume and end-diastolic volume in the ICU are not based on conductance pressure-volume catheter in the ventricle, but on echography. The ICU data are thus “one-shot” measurements and might be less accurate. Therefore, the optimal solution would be to validate the mathematical model using true clinical data obtained during weaning tests in the ICU.

## 5. Conclusions and Future Works

In this study, a mathematical model of the cardiovascular system connected to a VA-ECLS has been built and used to simulate weaning tests of a VA-ECLS.

The model is subject-specific, and the values of the different parameters have been estimated using experimental measurements. The validation of the mathematical model has been performed in two different configurations. One using no extracorporeal support (baseline situation) and one during weaning tests of a VA-ECLS. For the weaning test, the experimental measurements are hemodynamic data related to several extracorporeal blood flows (*Q*_d_).

The errors between the simulated values and the experimental data are negligible for the baseline situation, and the errors are small for most quantities (≤3%) during weaning tests. The slightly larger error on the stroke volume (≤5%) remains acceptable since it is related to the rather poor precision of the corresponding measurement.

We have shown that in the case of our experiments, for which a cardiac arrest was induced, the model and the identified contractility of the heart (*E*_h_) and systemic resistance (*R*_s_) can correctly describe the hemodynamic status of the animals. Moreover, we have proved that our cardiovascular model is able to nicely describe weaning tests from a VA-ECLS in pig experiments since the hemodynamic status for *Q*_d_ = 0 can be correctly predicted in silico.

Our work can thus be considered a proof of concept that a mathematical model could be very useful to help ICU clinicians to develop safer VA-ECLS weaning protocols that avoid unnecessary low values of the extracorporeal blood flow, which are associated with a higher risk of clotting.

The first important extension of the present work will consist in showing that our model can also be identified using a sufficient number of data corresponding to rather large values of the extracorporeal blood flow. Then, the model and approach will also require a validation using actual ICU data instead of animal data obtained in the case of artificially induced pathologies.

## Figures and Tables

**Table 1:** Parameters of the cardiovascular model connected to a VA-ECLS.

	Descriptions	Values
Identified parameters		
*A*_h_	Exponential parameter of the EDPVR equation	Subject-specific
*E*_a_	Elastance of the arteries	Subject-specific
*E*_h_	Slope of the ESPVR equation	Subject-specific
*R*_s_	Systemic resistance	Subject-specific
SBV	Stressed blood volume	Subject-specific
Measured parameters		
HR	Heart rate	Subject-specific
Parameters taken from the literature		
*B*_h_	Preexponential parameter of the EDPVR equation	0.35 mmHg [6]
*E*_v_	Elastance of the veins	0.014 mmHg/ml [6]
*R*_i_	Resistance of the input valve	0.015 mmHg·s/ml [6]
*R*_o_	Resistance of the output valve	0.03 mmHg·s/ml [6]
ECLS settings		
*Q*_d_	Extracorporeal blood flow	Chosen by the clinicians

**Table 2:** The different measurement standard errors.

Measurement	*σ*
max_T_(*V*_h_)	2 ml
PP_a_	0.4 mmHg
${\overset{¯}{P}}_{\text{a}}$	0.2 mmHg
${\overset{¯}{P}}_{\text{v}}$	0.1 mmHg
SV	3 ml

**Table 3:** Values of the physical parameters **p** and of the heart rate during the baseline situation. There is no noradrenaline injection for this state.

Data	Pig1	Pig2	Pig3	Pig4	Pig5	Pig6	Pig7	Pig8	Mean ± *σ*
Parameter identification									
*E*_h_ (mmHg/ml)	1.17	1.15	1.24	1.74	1.75	1.25	2.37	1.58	1.53 ± 0.42
SBV (ml)	846	1046	1064	1764	691	1093	1085	961	1069 ± 314
*A*_h_ (ml^−1^)	0.025	0.052	0.046	0.035	0.035	0.032	0.054	0.031	0.039 ± 0.011
*E*_a_ (mmHg/ml)	0.82	1.47	1.72	0.83	1.70	1.40	2.03	1.59	1.44 ± 0.43
*R*_s_ (mmHg·s/ml)	0.93	1.58	1.62	0.91	2.05	1.63	2.21	1.83	1.59 ± 0.47
Heart rate measurement									
HR (s^−1^)	1.26	1.18	1.35	1.69	1.17	1.23	1.31	1.33	1.32 ± 0.17

**Table 4:** Values of the coefficients **c** of the linear expressions for each parameter (*E*_h_, SBV, *A*_h_, *E*_a_, and *R*_s_). The value of the noradrenaline injection and the number of *Q*_d_ are also provided for each weaning test.

Data	Pig1	Pig2		Pig3	Pig4		Pig5		Pig6		Pig7		Pig8		Mean ± *σ*
	T1	T1	T2	T1	T1	T2	T1	T2	T1	T2	T1	T2	T1	T2	
Parameter identification															
*E*_0,h_ (mmHg/ml)	0.51	0.92	1.20	0.98	1.00	1.33	1.10	0.97	1.24	1.35	0.81	0.73	0.93	0.85	0.99 ± 0.24
SBV_0_ (ml)	783	723	613	1173	2105	1820	1279	1280	991	978	845	805	1016	970	1099 ± 419
*A*_0,h_ (ml^−1^)	0.026	0.059	0.047	0.041	0.031	0.032	0.040	0.040	0.037	0.036	0.030	0.030	0.027	0.026	0.036 ± 0.009
*E*_0,*a*_ (mmHg/ml)	0.63	1.34	1.22	1.22	1.72	2.11	1.73	1.03	1.80	1.53	1.70	1.67	1.62	1.48	1.49 ± 0.37
*R*_0,*s*_ (mmHg·s/ml)	0.96	1.74	1.44	1.00	0.90	1.15	1.21	1.26	1.21	0.98	1.57	1.58	1.55	1.41	1.28 ± 0.27
*E*_Sl,h_ (mmHg · s/ml^2^)	0.004	0.013	0.014	0.029	0.002	0.004	0.023	0.033	0.033	0.006	0.015	0.020	0.005	0.008	0.015 ± 0.011
SBV_Sl_ (s)	-0.44	6.09	-1.06	8.24	-0.852	-2.832	7.322	-6.923	11.748	8.577	3.180	1.954	4.609	1.696	2.081 ± 5.587
*A*_Sl,h_ (s/ml^2^)	‐2 · *e*‐4	‐6 · *e*‐5	3 · *e*‐4	‐2 · *e*‐4	5 · *e*‐5	3 · *e*‐5	9 · *e*‐5	1 · *e*‐6	1 · *e*‐4	1 · *e*‐5	‐1 · *e*‐4	‐1 · *e*‐4	6 · *e*‐5	4 · *e*‐5	‐9 · *e*‐6 ± 1 · *e*‐4
*E*_Sl,a_ (mmHg · s/ml^2^)	0.002	0.014	0.016	0.014	0.001	-0.008	0.011	0.065	-0.006	0.015	-0.023	-0.007	-0.011	0.000	0.006 ± 0.021
*R*_Sl,s_ (mmHg · s^2^/ml^2^)	-0.003	-0.034	-0.013	-0.009	-0.001	-0.016	0.005	0.016	-0.025	-0.001	-0.033	-0.030	-0.023	-0.014	‐0.013 ± 0.015
Noradrenaline															
Noradrenaline (*μ*g/min)	0.0	5.33	5.33	2.67	5.33	5.33	0.0	2.67	2.67	2.67	0.0	0.0	0.0	0.0	2.29 ± 0.2.29
Number of *Q*_d_															
*n*	3	3	3	3	5	6	5	5	5	5	5	5	8	9	

**Table 5:** Mean ± standard deviation of the errors between the simulations and the measurements of ${\overset{¯}{P}}_{\text{a}}$, PP_a_, ${\overset{¯}{P}}_{\text{v}}$, max_T_(*V*_h_), and SV for all weaning tests.

Error	Mean ± *σ*
Ψ(**c**^∗^)	149.9 ± 164.7
$\overset{¯}{\left|\Delta \text{P}{\text{P}}_{\text{a}}/\text{P}{\text{P}}_{\text{a}}\right|}$	0.025 ± 0.025
$\overset{¯}{\left|\Delta {\overset{¯}{P}}_{\text{a}}/{\overset{¯}{P}}_{\text{a}}\right|}$	0.016 ± 0.017
$\overset{¯}{\left|\Delta {\overset{¯}{P}}_{\text{v}}/{\overset{¯}{P}}_{\text{v}}\right|}$	0.014 ± 0.005
$\overset{¯}{\left|\frac{\Delta \text{ma}{\text{x}}_{\text{T}}\left(V_{\text{h}}\right)}{\text{ma}{\text{x}}_{\text{T}}\left(V_{\text{h}}\right)}\right|}$	0.009 ± 0.009
$\overset{¯}{\left|\Delta \text{SV}/\text{SV}\right|}$	0.046 ± 0.027
$\overset{¯}{\Delta \text{P}{\text{P}}_{\text{a}}/\text{P}{\text{P}}_{\text{a}}}$	‐0.003 ± 0.026
$\overset{¯}{\Delta {\overset{¯}{P}}_{\text{a}}/{\overset{¯}{P}}_{\text{a}}}$	0.008 ± 0.017
$\overset{¯}{\Delta {\overset{¯}{P}}_{\text{v}}/{\overset{¯}{P}}_{\text{v}}}$	0.011 ± 0.005
$\overset{¯}{\frac{\Delta \text{ma}{\text{x}}_{\text{T}}\left(V_{\text{h}}\right)}{\text{ma}{\text{x}}_{\text{T}}\left(V_{\text{h}}\right)}}$	0.002 ± 0.002
$\overset{¯}{\Delta SV/SV}$	0.010 ± ‐0.027

**Table 6:** Errors between the simulations and the measurements of ${\overset{¯}{P}}_{\text{a}}$, PP_a_, ${\overset{¯}{P}}_{\text{v}}$, max_T_(*V*_h_), and SV for each weaning test.

Data	Pig1	Pig2		Pig3	Pig4		Pig5		Pig6		Pig7		Pig8		Mean ± *σ*
	T1	T1	T2	T1	T1	T2	T1	T2	T1	T2	T1	T2	T1	T2	
Ψ(**p**^∗^)	47.5	31.4	507.9	231.9	82.2	501.7	219.0	104.3	24.2	10.0	22.9	125.6	91.8	97.5	149.9 ± 164.7
$\overset{¯}{\left|\Delta \text{P}{\text{P}}_{\text{a}}/\text{P}{\text{P}}_{\text{a}}\right|}$	0.019	0.018	0.092	0.068	0.007	0.008	0.021	0.030	0.008	0.006	0.016	0.031	0.009	0.011	0.025 ± 0.025
$\overset{¯}{\left|\Delta {\overset{¯}{P}}_{\text{a}}/{\overset{¯}{P}}_{\text{a}}\right|}$	0.023	0.019	0.066	0.030	0.005	0.010	0.024	0.012	0.005	0.002	0.004	0.017	0.007	0.006	0.016 ± 0.017
$\overset{¯}{\left|\Delta {\overset{¯}{P}}_{\text{v}}/{\overset{¯}{P}}_{\text{v}}\right|}$	0.016	0.020	0.010	0.014	0.014	0.015	0.018	0.004	0.015	0.009	0.011	0.014	0.021	0.019	0.014 ± 0.005
$\overset{¯}{\left|\Delta \text{ma}{\text{x}}_{\text{T}}\left(V_{\text{h}}\right)/\text{ma}{\text{x}}_{\text{T}}\left(V_{\text{h}}\right)\right|}$	0.006	0.009	0.010	0.014	0.014	0.040	0.008	0.006	0.005	0.003	0.001	0.005	0.004	0.006	0.009 ± 0.009
$\overset{¯}{\left|\Delta \text{SV}/\text{SV}\right|}$	0.009	0.055	0.097	0.081	0.062	0.084	0.042	0.054	0.016	0.021	0.020	0.037	0.033	0.032	0.046 ± 0.027
$\overset{¯}{\Delta \text{P}{\text{P}}_{\text{a}}/\text{P}{\text{P}}_{\text{a}}}$	0.005	0.003	-0.092	0.003	0.007	0.008	0.001	0.003	0.008	0.001	0.002	0.002	0.006	0.001	‐0.003 ± 0.026
$\overset{¯}{\Delta {\overset{¯}{P}}_{\text{a}}/{\overset{¯}{P}}_{\text{a}}}$	0.006	0.006	0.066	0.008	0.005	0.005	0.002	0.002	0.004	0.001	0.000	0.000	0.005	0.000	0.008 ± 0.017
$\overset{¯}{\Delta {\overset{¯}{P}}_{\text{v}}/{\overset{¯}{P}}_{\text{v}}}$	0.016	0.015	0.010	0.014	0.014	0.015	0.014	0.002	0.015	0.004	0.011	0.013	0.016	0.001	0.011 ± 0.005
$\overset{¯}{\Delta \text{ma}{\text{x}}_{\text{T}}\left(V_{\text{h}}\right)/\text{ma}{\text{x}}_{\text{T}}\left(V_{\text{h}}\right)}$	0.006	0.004	0.002	0.004	0.003	0.004	0.003	0.001	0.004	0.001	0.000	-0.001	0.004	0.000	0.002 ± 0.002
$\overset{¯}{\Delta \text{SV}/\text{SV}}$	0.005	0.009	-0.097	0.008	0.000	0.000	-0.002	-0.031	0.002	-0.002	-0.007	-0.006	0.002	-0.021	-0.010 ± 0.027
*n*	3	3	3	3	5	6	5	5	5	5	5	5	8	9	

**Table 7:** Values of the 8 coefficients and of the “normalized” variation of the parameters.

Identified coefficients	
*E*_0,h_	0.905 (mmHg/ml)
SBV_0_	769 (ml)
*A*_0,h_	0.035 (ml^−1^)
*R*_0,s_	0.356 (mmHg·s/ml)
*E*_Sl,h_	0.0034 (mmHg·s/ml^2^)
SBV_Sl_	1.44 (s)
*A*_Sl,h_	4 · *e*‐5 (s/ml^2^)
*R*_Sl,s_	0.0020 (mmHg·s^2^/ml^2^)
Variation of the 4 physiological parameters	
Δ_1_*E*_h_/*E*_h_	0.06
Δ_1_SBV/SBV	0.03
Δ_1_*A*_h_/*A*_h_	0.02
Δ_1_*R*_s_/*R*_s_	0.09

## Data Availability

All experimental data used in this study are available from the corresponding author upon request.

## Appendix

### A. Mathematical Modeling

#### A.1. Elastic Chambers

The arteries and veins are described by

(A.1) $$\begin{array}{c} P_{\text{a}}\left(t\right)=E_{\text{a}}\cdot V_{\text{s},\text{a}}\left(t\right),\\ P_{\text{v}}\left(t\right)=E_{\text{a}}\cdot V_{\text{s},\text{v}}\left(t\right). \end{array}$$

On the other hand, the heart is represented using the description of Burkhoff and Tyberg [6]:

(A.2) $$\begin{array}{c} P_{\text{h}}\left(t\right)=e\left(t\right)\cdot P_{\text{es}}\left(t\right)+\left(1-e\left(t\right)\right)\cdot P_{\text{ed}}\left(t\right) \end{array}$$

where

- *P*_es_(*t*) = *E*_h_ · *V*_s,h_(*t*) is the end-systolic pressure
- *P*_ed_(*t*) = *B*_h_ · (*e*^*A*_h_·*V*_s,h_(*t*)^ − 1) is the end diastolic pressure
- *e*(*t*) is the driver function, and several formula have been proposed in literature [6, 24, 25]

### B. Vessels

The systemic blood flow *Q*_s_ is described by

(B.1) $$\begin{array}{c} Q_{\text{s}}\left(t\right)=\frac{P_{\text{a}}\left(t\right)-P_{\text{v}}\left(t\right)}{R_{\text{s}}}. \end{array}$$

The blood flows crossing the valves are different since the cardiac valves allow the blood flow to circulate in only one direction. The blood flows *Q*_i_ and *Q*_o_ are thus described by

(B.2) $$\begin{array}{c} Q_{\text{i}}\left(t\right)=\left\{\begin{array}{cc} 0, & \text{if }P_{\text{v}}\left(t\right)\leq P_{\text{h}}\left(t\right),\\ \frac{P_{\text{v}}\left(t\right)-P_{\text{h}}\left(t\right)}{R_{\text{i}}}, & \text{if }P_{\text{v}}\left(t\right)>P_{\text{h}}\left(t\right), \end{array}\right.\\ Q_{\text{o}}\left(t\right)=\left\{\begin{array}{cc} 0, & \text{if }P_{\text{h}}\left(t\right)\leq P_{\text{a}}\left(t\right),\\ \frac{P_{\text{h}}\left(t\right)-P_{\text{a}}\left(t\right)}{R_{\text{o}}}, & \text{if }P_{\text{h}}\left(t\right)>P_{\text{a}}\left(t\right). \end{array}\right.\; \end{array}$$

### C. Mass Balance Equations

The variations of the volume of an elastic chamber ${\dot{V}}_{\text{s}}$ is equal to the difference between flows coming into and going out of this chamber. ${\dot{V}}_{\text{s},\text{h}}$, ${\dot{V}}_{\text{s},\text{a}}$, and ${\dot{V}}_{\text{s},\text{v}}$ are thus described by

(C.1) $$\begin{array}{c} {\dot{V}}_{\text{s},\text{h}}=Q_{\text{i}}-Q_{\text{o}}, \end{array}$$

(C.2) $$\begin{array}{c} {\dot{V}}_{\text{s},\text{a}}=Q_{\text{o}}-Q_{\text{s}}+Q_{\text{d}}, \end{array}$$

(C.3) $$\begin{array}{c} {\dot{V}}_{\text{s},\text{v}}=Q_{\text{s}}-Q_{\text{i}}-Q_{\text{d}}. \end{array}$$

If equations (C.1–C.3) are summed up, the following equation is obtained:

(C.4) $$\begin{array}{c} {\dot{V}}_{\text{s},\text{h}}+{\dot{V}}_{\text{s},\text{a}}+{\dot{V}}_{\text{s},\text{v}}=0. \end{array}$$

The right-hand side of equation (C.4) is equal to 0, and this confirms that the system is a closed loop. The integration of both-hand side of equation (C.4) gives the following relation:

(C.5) $$\begin{array}{c} V_{\text{s},\text{h}}+V_{\text{s},\text{a}}+V_{\text{s},\text{v}}=\text{SBV}. \end{array}$$
